# Proteomic analysis of laser capture microdissected focal lesions in a rat model of progenitor marker-positive hepatocellular carcinoma

**DOI:** 10.18632/oncotarget.15219

**Published:** 2017-02-09

**Authors:** Adeola O. Adebayo Michael, Nagib Ahsan, Valerie Zabala, Heather Francois-Vaughan, Stephanie Post, Kate E. Brilliant, Arthur R. Salomon, Jennifer A. Sanders, Philip A. Gruppuso

**Affiliations:** ^1^ Department of Pediatrics, Rhode Island Hospital and Brown University, Providence, RI, USA; ^2^ Division of Biology and Medicine, Brown University, Providence, RI, USA; ^3^ Center for Cancer Research Development, Proteomics Core Facility, Rhode Island Hospital, Providence, RI, USA; ^4^ Department of Environmental and Evolutionary Biology, Brown University, Providence, RI, USA; ^5^ Department of Molecular Biology, Cell Biology and Biochemistry, Brown University, Providence, RI, USA; ^6^ Department of Pathology and Laboratory Medicine, Brown University, Providence, RI, USA; ^7^ Current address: Department of Pathology, University of Pittsburgh, Pittsburgh, PA, USA

**Keywords:** hepatocellular carcinoma, liver, microdissection, proteomics, pre-neoplasia

## Abstract

We have shown previously that rapamycin, the canonical inhibitor of the mechanistic target of rapamycin (mTOR) complex 1, markedly inhibits the growth of focal lesions in the resistant hepatocyte (Solt-Farber) model of hepatocellular carcinoma (HCC) in the rat. In the present study, we characterized the proteome of persistent, pre-neoplastic focal lesions in this model. One group was administered rapamycin by subcutaneous pellet for 3 weeks following partial hepatectomy and euthanized 4 weeks after the cessation of rapamycin. A second group received placebo pellets. Results were compared to unmanipulated control animals and to animals that underwent an incomplete Solt-Farber protocol to activate hepatic progenitor cells. Regions of formalin-fixed, paraffin-embedded tissue were obtained by laser capture microdissection (LCM). Proteomic analysis yielded 11,070 unique peptides representing 2,227 proteins. Quantitation of the peptides showed increased abundance of known HCC markers (e.g., glutathione S-transferase-P, epoxide hydrolase, 6 others) and potential markers (e.g., aflatoxin aldehyde reductase, glucose 6-phosphate dehydrogenase, 10 others) in foci from placebo-treated and rapamycin-treated rats. Peptides derived from cytochrome P450 enzymes were generally reduced. Comparisons of the rapamycin samples to normal liver and to the progenitor cell model indicated that rapamycin attenuated a loss of differentiation relative to placebo. We conclude that early administration of rapamycin in the Solt-Farber model not only inhibits the growth of pre-neoplastic foci but also attenuates the loss of differentiated function. In addition, we have demonstrated that the combination of LCM and mass spectrometry-based proteomics is an effective approach to characterize focal liver lesions.

## INTRODUCTION

Hepatocellular carcinoma (HCC) is the most common type of liver cancer [1]. HCC carries a grave prognosis due to the often advanced nature of the disease at time of diagnosis that is, in turn, a consequence of the unavailability of sensitive and specific diagnostic markers [1]. The identification of suitable, early diagnostic markers could identify individuals for timely and effective clinical treatment, and may be useful in the identification of individuals at high risk of developing HCC. Previous studies in our laboratory showed that the canonical signaling pathway involving the mechanistic target of rapamycin (mTOR) and, more specifically, mTOR complex 1 (mTORC1), is activated during the early stages of focal lesion development in the Solt-Farber model of progenitor marker-positive HCC [2, 3]. We showed that rapamycin, the prototypical mTORC1 inhibitor, affected a broad spectrum of genes associated with the development of persistent focal lesions such that short-term mTORC1 inhibition resulted in a genetic signature more reminiscent of normal liver [3].

While analyses of the transcriptome using gene array profiling has identified numerous genes that may contribute to focal lesion development and HCC progression in animal models and in humans [4–10], a marked divergence can occur between mRNA transcript level and protein abundance [11]. This is particularly the case in a highly complex and heterogeneous microenvironment such as that seen in HCC [12]. We therefore decided to undertake a global proteomic analysis of persistent lesions and other relevant samples from the same model system.

Formalin-fixed, paraffin-embedded (FFPE) tissue samples offer the opportunity to correlate molecular data with pathological characterization and, therefore, a relation of protein biomarkers to disease state [13]. Fresh or frozen tissue samples, although ideal, are difficult to use and store in large numbers. FFPE tissues provide an alternative to frozen tissue for conducting proteomic investigations [14–16]. They are inherently stable at room temperature, more widely available, and suitable for long-term storage. In addition, they are compatible for use with laser capture microdissection (LCM) technology [17]. Recent years have seen improved methods to reverse nucleic acid and protein crosslinking in order to obtain DNA, RNA and protein for high-throughput studies [18–21]. Coupled with the development of mass spectrometers with greater sensitivity and resolution, this approach can complement genomic analysis and offer insights that can be gained from a network biology analysis [22].

In the present study, we used LCM, a protein extraction protocol, and liquid chromatography-tandem mass spectrometry (LC-MS/MS) coupled with label-free peptide quantitation to identify and quantify peptides from FFPE tissue sections. We applied these methods to the characterization of focal lesions induced using the Solt-Farber rat model of hepatocellular carcinoma (11, 35). In this model, rats are administered a single dose of a carcinogen, diethylnitrosamine (DENA), followed 14 days later by a single dose of 2-acetylaminofluorene (2-AAF), an agent that suppresses normal hepatic proliferation through formation of DNA adducts (14). Three weeks after DENA administration, a 2/3 partial hepatectomy is performed to stimulate hepatocyte proliferation. Rats were euthanized 7 weeks after partial hepatectomy. We compared the resulting pre-neoplastic foci to normal liver and to a model in which rats were administered 2-AAF followed by 2/3 partial hepatectomy. This protocol, sometimes referred to as the incomplete Solt-Farber model, suppresses the proliferation of hepatocytes resulting in the proliferation of hepatic progenitor cells in response to partial hepatectomy (11). Given our prior observation regarding the role of mTORC1 in the Solt-Farber model [3], we also characterized persistent focal lesions from rats administered rapamycin during the three weeks following partial hepatectomy. The results of our study showed that rapamycin is not only a potent inhibitor of focal lesion growth, but that it has a salutary effect on the focal lesion phenotype. Furthermore, while the Solt-Farber protocol produces neoplastic lesions that are progenitor marker-positive, proteomic analysis of the focal lesions did not show evidence for persistent progenitor cells, consistent with the transitional nature of these earlier lesions [8]. Finally, our study supports the use of combined LCM and LC-MS/MS to characterize focal liver abnormalities.

## RESULTS

### Peptide yield, reproducibility and results across samples

**Figure 1:**
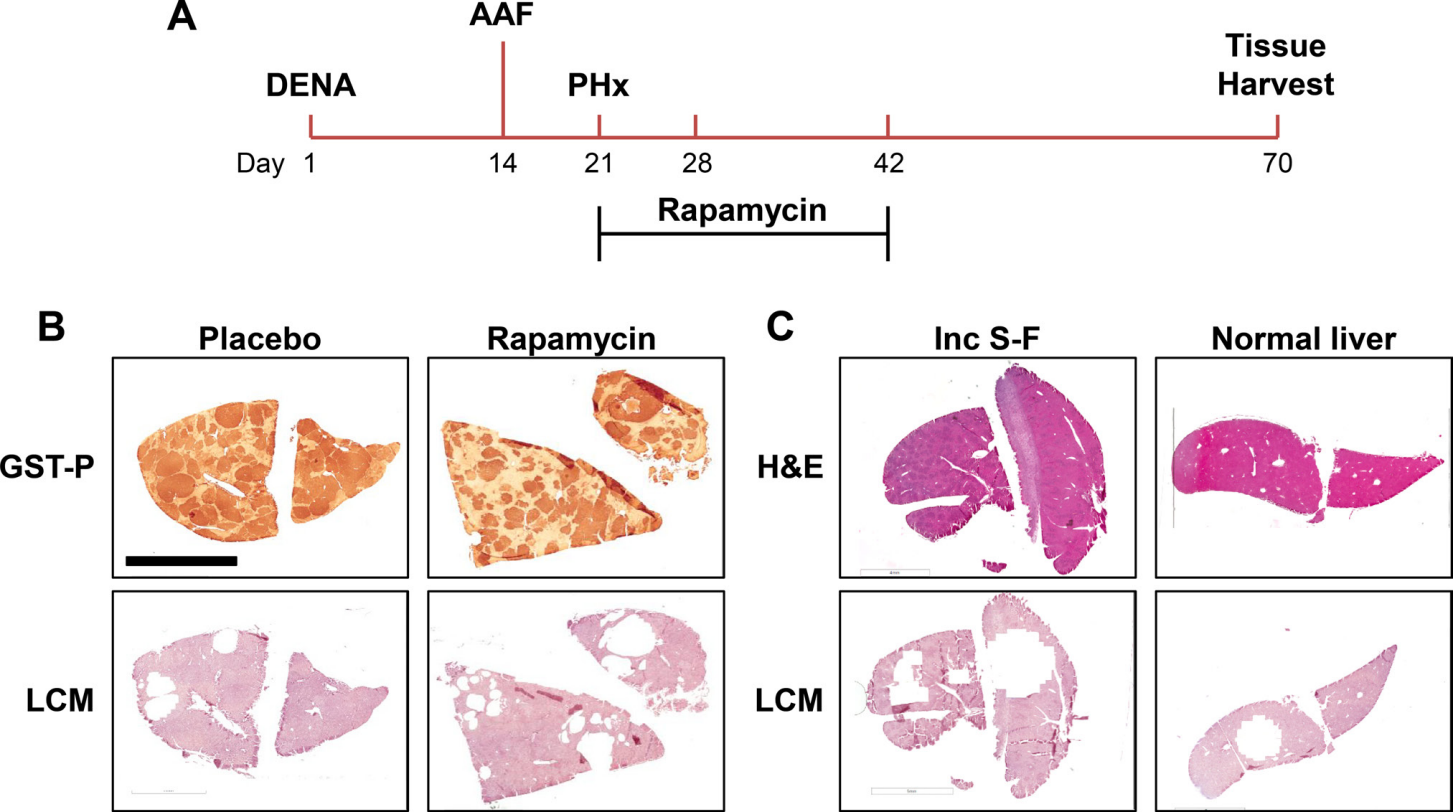
Experimental design (**A**) The time line for the Solt-Farber experimental protocol begins with a single injection of DENA. AAF is administered by a time-release intraperitoneal pellet over one week leading up to the 2/3 partial hepatectomy. Animals were divided into two experimental groups. One received rapamycin for 3 weeks by time-release subcutaneous pellet. The second group was implanted with placebo pellets. (**B**) Laser capture microscopy (LCM) was used to obtain preneoplastic foci. Adjacent sections were stained for GST-P to verify the selection of foci tissue. (**C**) LCM was similarly employed to select areas from the incomplete Solt-Farber (Inc S-F) model and normal liver tissue. The scale bar in the upper left image of panel B represents 5 mm.

Our experimental protocol for inducing pre-neoplastic lesions and treating with rapamycin is summarized in Figure 1A. We studied three animals per condition. Four technical replicates were analyzed per animal. Representative images demonstrating LCM and the selection of specific tissue regions for all four experimental groups are shown in Figure 1B and 1C. The region captured from the incomplete Solt-Farber protocol (Figure 1C) contains a mix of oval cells interspersed with 2-AAF-suppressed hepatocytes.

Inspection of the proteomic data across all 48 samples (Supplementary Table 1) showed the identification of 11,070 unique peptide sequences representing 2,227 unique proteins. The number of unique peptides identified for each of the 48 analyses ranged from a low of 7,525 to a high of 10,879. The low figure was an outlier (an incomplete Solt-Farber technical replicate) with the next lowest peptide yield being 10,472. The corresponding number of unique proteins ranged from 1,655 (next lowest was 2,115) to 2,200.

Approximately 55% of the peptides detected in any of the 48 samples were detected in all 48 samples; 89% of the peptides were detected in at least 43 (90%) of the 48 samples. Combining technical replicates, 95% of peptides were detected and quantified in all three biological replicates for the four experimental groups, indicating that the LCM and peptide preparation protocols were reproducible. To assess the degree of variance among analyses, we examined the coefficient of variation (COV) for technical replicates within biological replicates and for biological replicates within experimental groups. In both cases, the mean and median COVs were in the range of 0.4–0.5 and were normally distributed.

**Figure 2:**
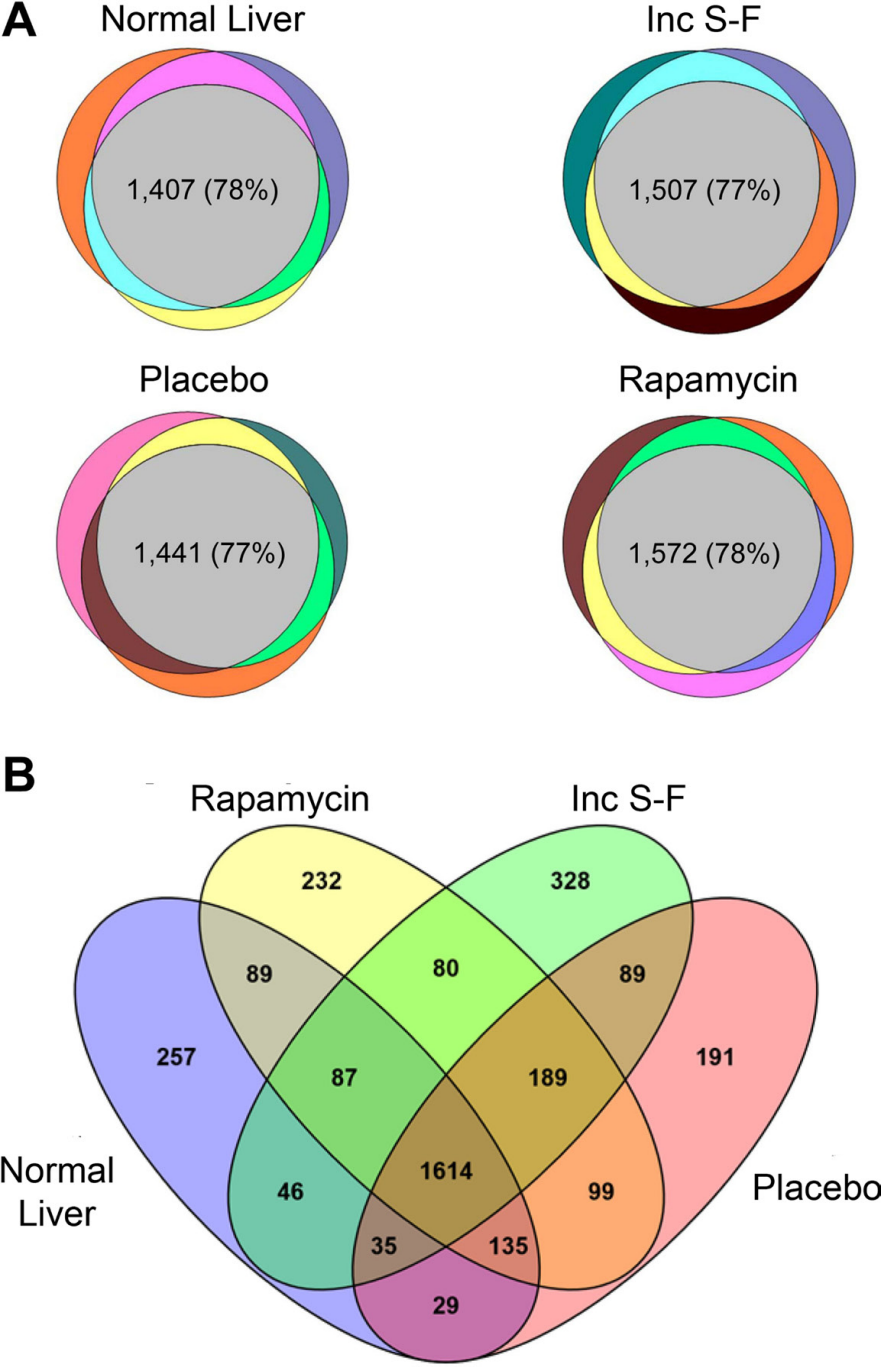
Venn diagrams showing the detection of peptides and proteins across biological replicates for each experimental group and for all experimental groups (**A**) shows the three biological replicates for each of the four experimental groups. The gray central area in each diagram depicts the number and percentage of peptides that were detected in all three replicates for each group. (**B**) shows peptides in common between the various experimental groups. The data are based on any proteins detected in any of the biological replicates for each group.

To further assess the consistency with which proteins were identified in biological replicates, technical replicate quantitative results were averaged and the proportion of peptides identified by MS/MS spectral assignment in one, two or three of the biological replicates within experimental groups was determined. For all four experimental groups, 77–78% of the peptides were identified in 3 of 3 biological replicates (Figure 2A). The proteins identified by at least one peptide in each of the experimental groups were 2,292 for normal liver, 2,381 for the placebo foci, 2,525 for the rapamycin foci and 2,468 for the incomplete Solt-Farber samples. Proteins common to all four experimental groups numbered 1,614 (Figure 2B). The overlap between the various groups was relatively consistent, ranging from 1,782 (common to the normal liver and incomplete Solt-Farber samples) to 2,037 (common to the placebo and rapamycin foci).

**Figure 3:**
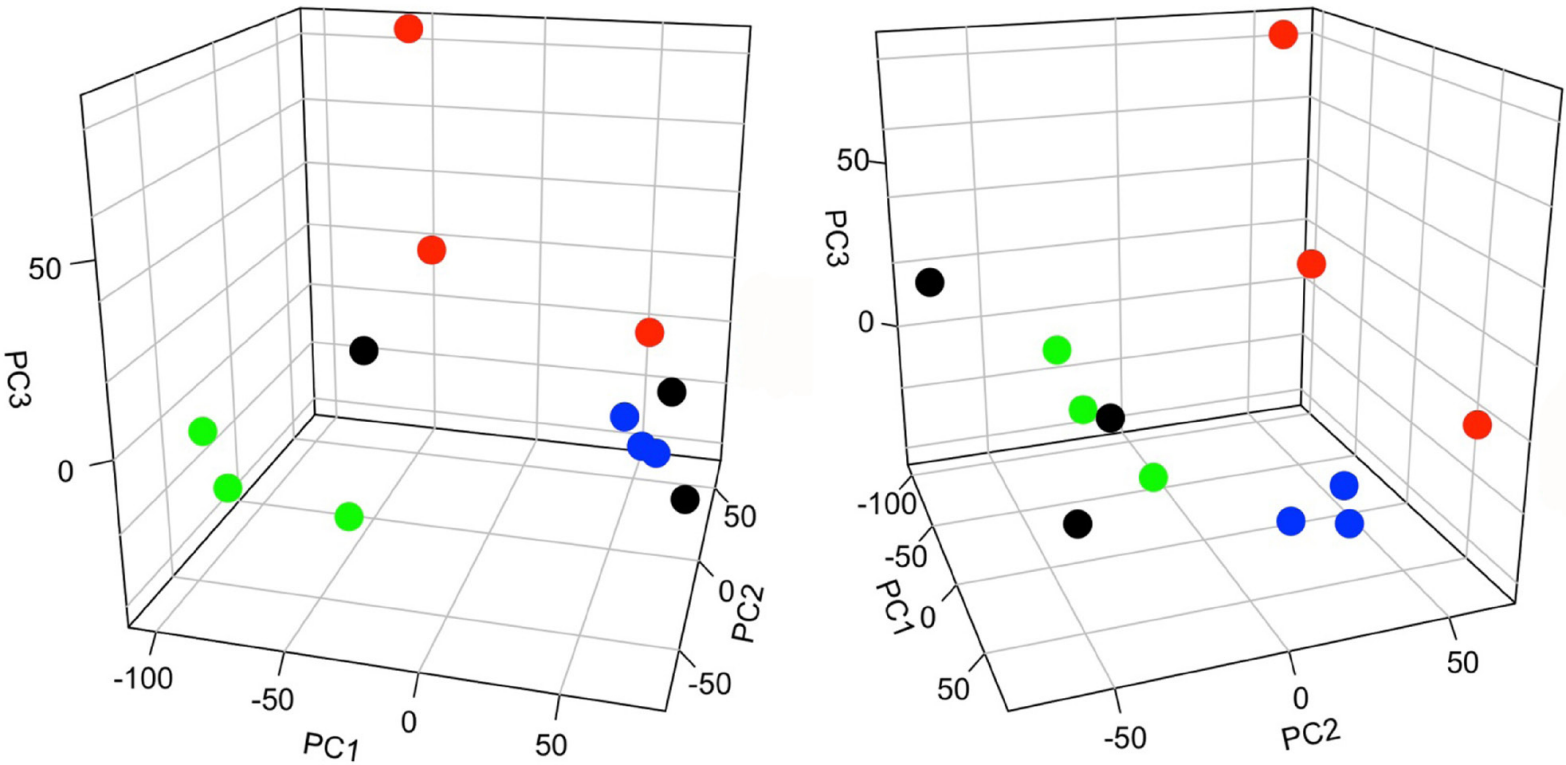
Principal component analysis (PCA) of peptide abundance data Two views of the same PCA plot are shown. The first, second, and third components (PC1, PC2, PC3) represent 40.2%, 16.4%, and 10.1% of the variance in the data, respectively. Control liver, black; incomplete Solt-Farber samples, green; Solt-Farber placebo group, red; Solt-Farber rapamycin group, blue.

Principal component analysis (PCA) was also used to assess similarities and differences between groups. Analyses were performed using the means of technical replicates for the three biological replicates per experimental group. Results showed discernable differences between all four groups (Figure 3). The Solt-Farber placebo group was segregated to a greater degree from control liver than was the Solt-Farber rapamycin group. The incomplete Solt-Farber group was more dissimilar from the two Solt-Farber groups than was control liver.

### Quantitative analysis of LC-MS/MS results

**Figure 4:**
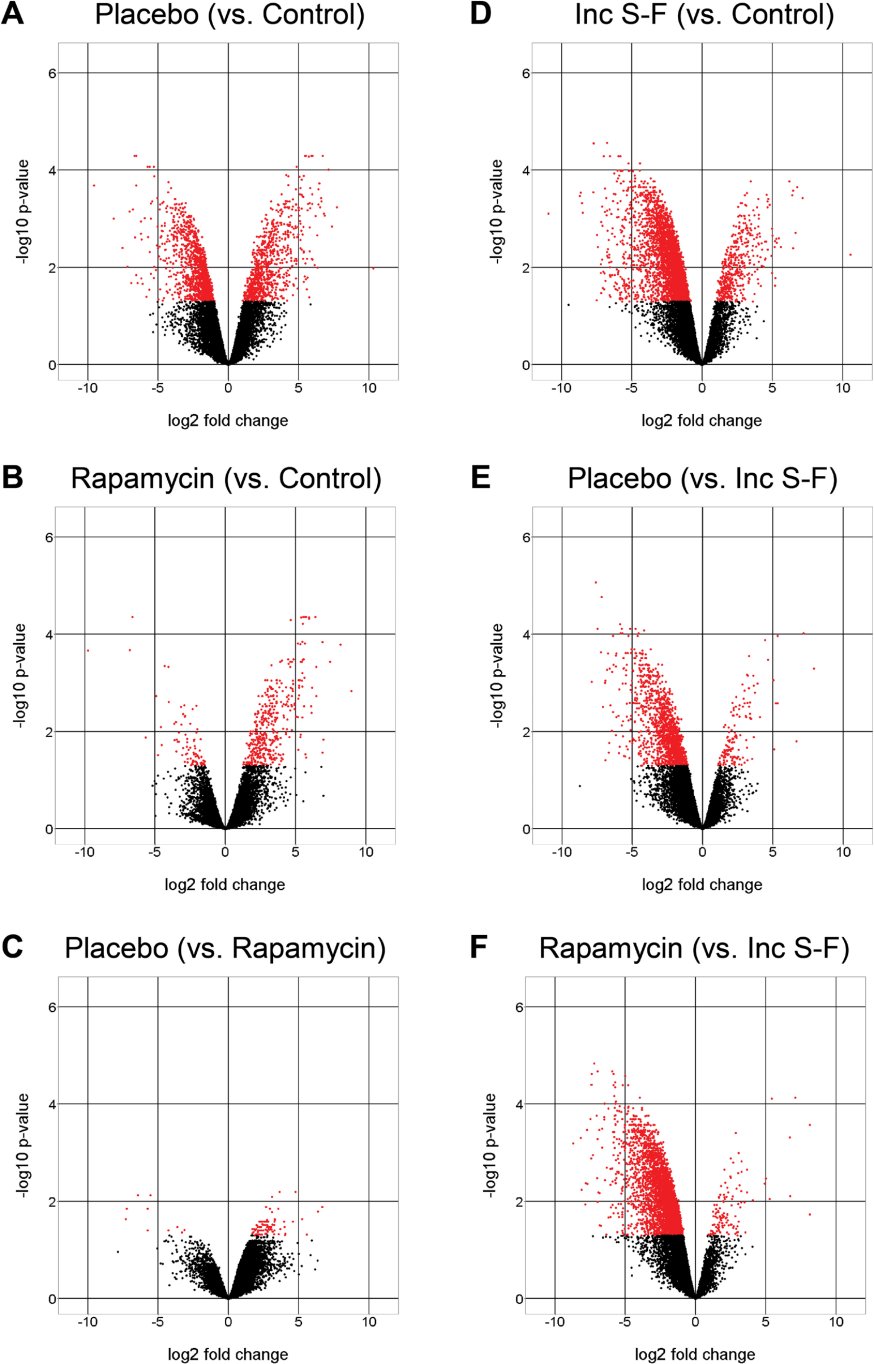
Volcano plots for six pair-wise analyses Comparisons are shown as the negative log_10_ of the *q*-value versus the log_2_ of the fold-differences between groups for liver peptide abundance. Red dots represent peptides for which the corrected *P-value* was below the assigned level of significance (0.05).

Analysis of variance (ANOVA) was used to identify peptides that showed significant differences between groups. The numbers of significant peptides and corresponding proteins for each of the six pair-wise analyses are given in Table 1. The full list of significant peptides along with protein identifications, fold-differences and corrected *P*-values are provided in Supplementary Table 2. We displayed the distribution of significant peptides based on fold-differences in abundance for the six analyses using volcano plots (Figure 4). Based on this depiction of the data, it appears that the rapamycin samples differ from control liver to a substantially lesser degree than the placebo samples (Figure 4A and 4B). This distinction appeared to be a result of far fewer peptides showing reduced abundance relative to control liver for the rapamycin samples compared to the placebo samples. Comparisons to the incomplete Solt-Farber group yielded the opposite result; that is, the rapamycin samples showed a greater degree of difference from the incomplete Solt-Farber samples than did the placebo samples (Figure 4E and 4F). These global differences between the placebo and rapamycin groups were not reflected in substantial differences between the placebo and rapamycin groups in a direct comparison of the two (Figure 4C). While the fold-difference was large, the degree of significance was small. This may reflect the considerable variance in both groups relative to the variance among the normal liver samples.

**Table 1:** Significant peptides and corresponding proteins identified by ANOVA and pair-wise post-hoc analysis

Comparison Groups	Significant Peptides	Corresponding Proteins
Placebo versus Control	2,248	895
Rapamycin versus Control	503	231
Placebo versus Rapamycin	97	85
Incomplete Solt-Farber versus Control	4,289	1,233
Placebo versus Incomplete Solt-Farber	1,684	599
Rapamycin versus Incomplete Solt-Farber	4,315	1,174

These comparisons led us to hypothesize that the rapamycin samples maintained a greater degree of functional differentiation than the placebo samples. We further hypothesized that the situation with the incomplete Solt-Farber model was the converse. That is, given the presence of a significant number of oval cells, the samples would show a loss of differentiated hepatic function. Thus, the greater degree of difference between the rapamycin group and incomplete Solt-Farber group again reflects the maintenance of a more well differentiated state in the rapamycin group.

### Pathway analysis

**Figure 5:**
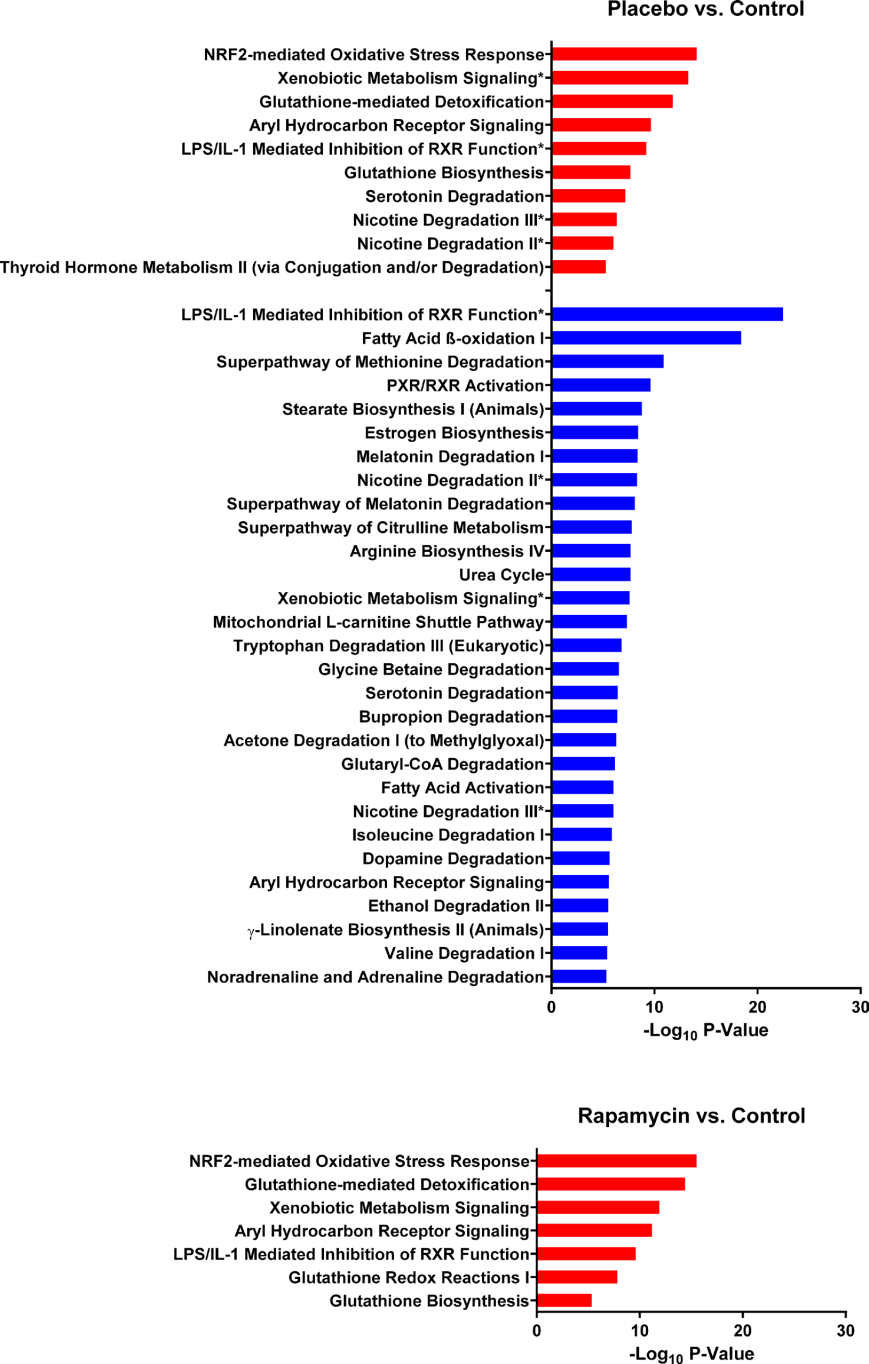
Canonical pathways that differ between the two Solt-Farber experimental groups and control liver The input for these comparisons were proteins represented by at least two peptides that were significantly different in the placebo-control or rapamycin-control analyses. Red bars represent pathways that were upregulated based on peptide abundance in the experimental group. Blue bars represent pathways that were downregulated in the experimental group. The adjusted *P-value* is shown as −log_10_ for all pathways significant at a level of *P* < 10^−5^. Asterisks denote pathways that were both upregulated and downregulated based on effects on different proteins.

To test the aforementioned hypotheses, we subjected the proteins corresponding to significantly different peptides to pathway analysis using Ingenuity Pathway Analysis (IPA^®^). A comparison of the two Solt-Farber groups, placebo and rapamycin, with control liver (Figure 5) illustrates the marked difference between these groups. While both showed upregulation of similar pathways relative to control liver, only the placebo group showed significant downregulation of pathways. These downregulated pathways represented, in general, differentiated liver functions, such as metabolic pathways for lipids, steroids and amino acids metabolism, as well as bile acid metabolism and ammonia detoxification. An examination of the proteins that accounted for these downregulated pathways (Supplementary Table 3) showed considerable overlap between pathways with cytochrome P450 enzymes predominating. In contrast, the pathways that were upregulated in the placebo group relative to normal liver were recapitulated in the rapamycin group. In both cases, glutathione transferases and aldehyde dehydrogenases predominated.

**Figure 6:**
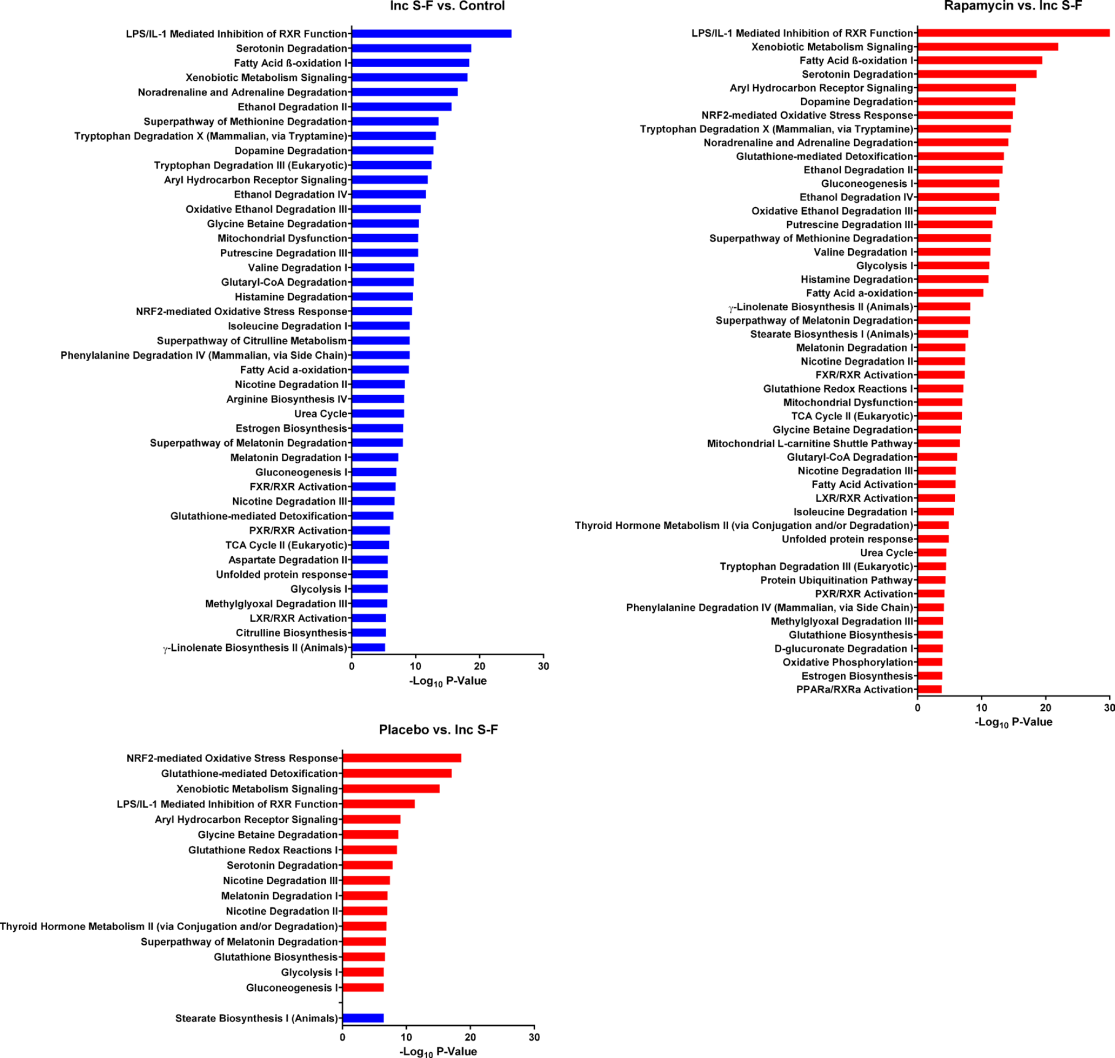
Canonical pathways that differ in comparisons using the incomplete Solt-Farber group (Inc S-F) The input for these comparisons was determined as for Figure 5 using incomplete Solt-Farber-control, placebo-incomplete Solt-Farber and rapamycin-incomplete Solt-Farber analyses. Red bars represent pathways that were upregulated based on peptide abundance in the experimental group. Blue bars represent pathways that were downregulated in the experimental group. The adjusted *P-value* is shown as −log_10_ for all pathways significant at a level of *P* < 10^−5^.

The incomplete Solt-Farber group showed downregulation of numerous pathways relative to normal liver (Figure 6). This observation was consistent with a loss of differentiated liver functions, including fatty acid metabolism, amino acid metabolism, and pathways involving cytochrome P450 enzymes. The proteins that accounted for these pathways (Supplementary Table 3) were, in many cases, aldehyde dehydrogenases, hydroxysteroid dehydrogenases and cytochrome P450 enzymes. Changes in the two Solt-Farber models (Figure 6) were largely restricted to increases in pathways related to glutathione metabolism and phase II drug conjugation reactions. The degree of significance for differences between the two Solt-Farber groups and the incomplete Solt-Farber samples was greater for the rapamycin group than it was for the placebo group.

The pathway analysis results support the interpretation that the rapamycin group more closely resembles normal liver than does the placebo group. Although there were few significant differences between the placebo and rapamycin groups, too few for pathway analysis, the former showed marked differences in relation to normal liver. The inverse being the case in comparisons with the incomplete Solt-Farber group is also consistent with the rapamycin samples having greater similarity to normal liver than the samples containing oval cells.

### Identification of known and potentially novel preneoplastic markers

In order to visualize our data and further assess the validity of our observations, we generated heat maps. Many of the peptides that differed significantly between groups represented proteins from which multiple peptides were derived. To examine the degree of consistency within our data, we calculated mean values for the three biological replicates by averaging the results of four technical replicates. Heat maps were constructed using the ratio of each biological sample to the mean of the three control group samples.

**Figure 7:**
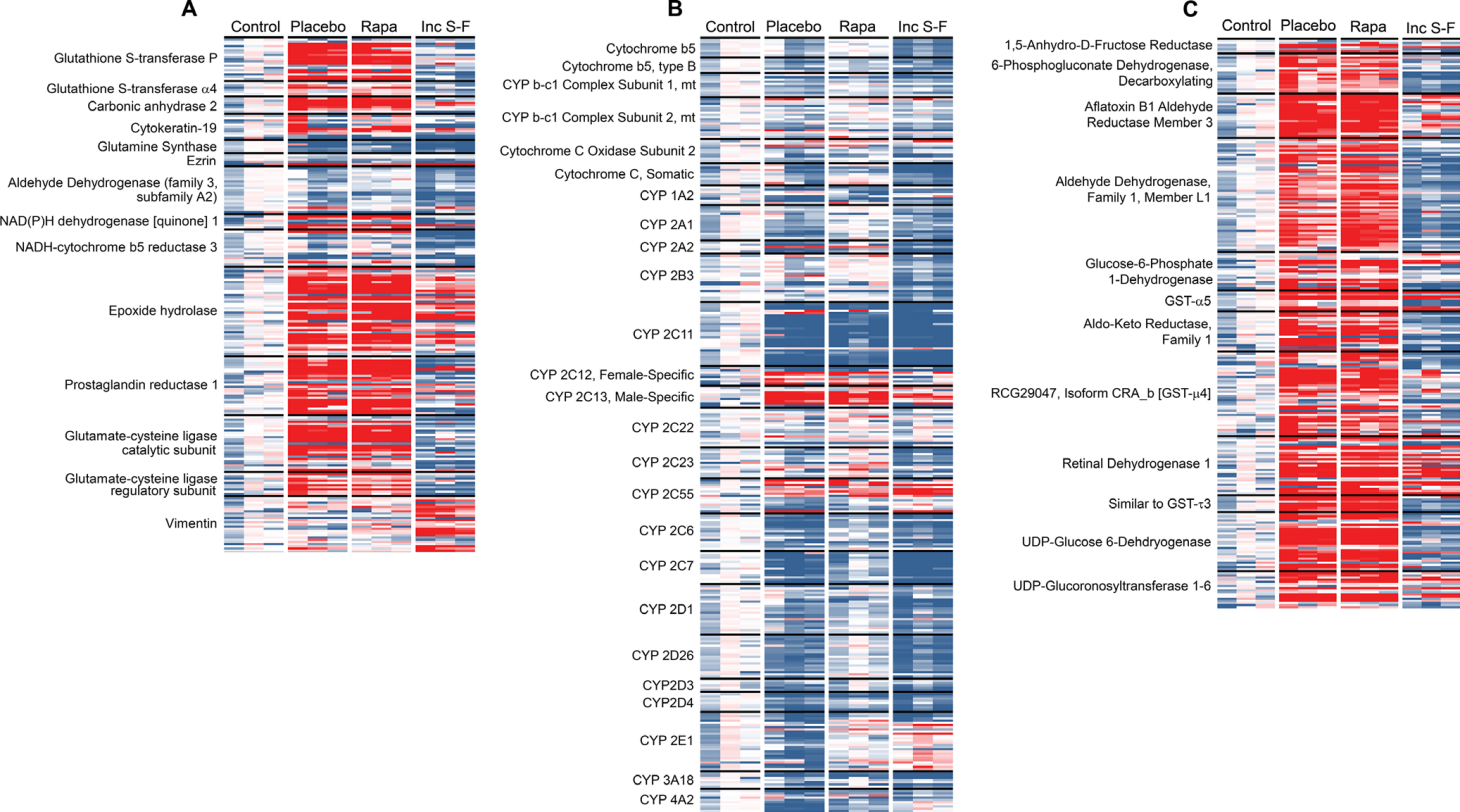
Heat maps showing the relative abundance of peptides in each of the twelve biological samples (**A**) shows peptides corresponding to known HCC markers. For (**B**), all peptides for cytochrome-related proteins were analyzed. (**C**) was constructed by generating a heat map for all remaining peptides, then extracting those that demonstrated consistent over-abundance in one or both of the Solt-Farber samples (placebo and rapamycin). For all three heat maps, each row represents one peptide. For each peptide, the mean of four technical replicates for each biological replicate was calculated. The result for each sample was expressed as a ratio to the mean of the three control samples. The colors range from deep blue for a ratio of 0.2 to bright red for a ratio of 5.0, with white assigned to a ratio of 1.0. Only proteins for which at least 5 peptides were quantified are shown in the figure.

We first constructed a heat map for proteins identified previously as overexpressed in HCC (Figure 7A). In some cases, these purported markers were identified in analyses of human HCC [23, 24] or hepatic cell lines [23]. However, our main focus was on markers previously identified in rodent studies [23–32] that used the Solt-Farber model or a modification thereof.

This analysis showed that some markers, such as glutathione S-transferase placental type (GST-P), epoxide hydrolase, prostaglandin reductase 1, and glutamate-cysteine ligase (catalytic and regulatory subunits) were indeed present at higher abundance in the focal preneoplastic lesions. Others, such as glutamine synthase, ezrin and aldehyde dehydrogenase (family 3, subfamily A2) were not present at higher abundance. Two oval cell markers, vimentin and cytokeratin-19, were represented by multiple, quantified peptides. The former was present in higher abundance in samples from the incomplete Solt-Farber model. That was not the case for the latter. Some peptides representing cytokeratin-19 appeared to be present in relatively high abundance in the focal lesions, although the results were insufficiently consistent to consider the focal lesions “progenitor cell marker-positive.” Vimentin-derived peptides were not present at high levels in the focal lesions. None of the markers included in this analysis showed an effect of rapamycin.

In a second focused analysis, we examined the relative levels of peptides associated with cytochrome P450 enzymes (CYPs; Figure 7B). There is an established literature showing a general reduction in the activities of P450 enzymes in hepatic nodules [33]. Consistent with this, we observed a general trend toward reduced peptide abundance in the placebo group foci relative to the control liver samples. There was a similar reduction in the incomplete Solt-Farber samples. The effect was less pronounced in the rapamycin group. A notable exception to the general effect across P450 enzymes was seen with P450 2C13, which showed an increase in both placebo group and rapamycin group foci. More modest and less consistent effects were seen for P450 2C12, which is referred to as female-specific, and P450 2C55.

We performed an additional analysis in which all remaining proteins represented by at least 5 peptides were examined for increased abundance in the preneoplastic foci (Figure 7C). The purpose of this analysis was to identify potential marker proteins. Twelve proteins were identified. In all cases, results were similar for the placebo and rapamycin groups. In most cases, results differed for the incomplete Solt-Farber samples. In fact, the peptide levels for 7 of these proteins tended to be lower in the incomplete Solt-Farber samples relative to the control samples.

## DISCUSSION

The goal of the present study was several-fold. We first aimed to establish methods that would allow us to use unlabeled quantitation of peptide abundance to characterize the proteome of focal liver lesions. One approach we used to validate the method was to examine the abundance of known protein markers associated with preneoplasia and HCC in rats as well as markers of human HCC. A corollary of this was the identification of new markers of preneoplastic foci in the Solt-Farber model. Our experimental design included the comparison with the incomplete Solt-Farber model of progenitor cell expansion, the purpose being two-fold; a control for the effects of 2-AAF and 2/3 partial hepatectomy on the parenchyma and the examination of preneoplastic foci for progenitor cell markers. Finally, the central aim of the present studies was to further characterize our model of short-term exposure to rapamycin during the early stage of preneoplastic foci development. Our aim was to answer the question of whether rapamycin, in potently inhibiting the growth of foci, is also inhibiting the progression of these lesions or, conversely, selecting for more dysplastic lesions.

Our characterization of the proteome of the focal lesions was successful in that it yielded a sufficient number of peptides to readily differentiate normal tissue from preneoplastic foci and from regions containing significant oval cells and AAF-suppressed hepatocytes from the incomplete Solt-Farber model. In all, peptides corresponding to 2,227 unique proteins were identified across the four experimental groups, with 95% of peptides quantified in all three biological replicates for each of the four experimental conditions. Volcano plots indicated clear differences between the three experimental groups and control liver. This approach to visualizing the data also indicated few significant peptides in a direct comparison of the placebo and rapamycin groups. We attributed this to high variance, given that mean fold-differences were similar to those seen in other comparisons. However, peptides identified as significant based on ANOVA and post-hoc pairwise analyses for other comparisons were great in number, representing nearly nine hundred proteins for the placebo-control comparison. Pathway analyses using the proteins corresponding to these significant peptides showed that the preponderance of the findings could be attributed to a relatively small number of functional categories.

These analyses were complimented by the visualization of our data using heat maps. This approach showed consistency among multiple peptides representing known pre-neoplastic and neoplastic markers. These were compiled through a literature search that included proteins identified through a variety of methods, including gene expression analyses, immunologic techniques and measurements of enzyme activities. We included data derived from both animal and human studies. Our data showed clear overabundance of some of these known markers, including GST-P, NAD(P)H dehydrogenase 1, epoxide hydrolase, prostaglandin reductase 1, carbonic anhydrase 2 and the regulatory and catalytic subunits of glutamate-cysteine ligase [10, 23, 25–30, 32]. A number of other markers were not identified as present in higher abundance based on peptide quantification. Of particular note were two oval cell markers, CK-19 and vimentin [34]. The latter appeared to be upregulated in the incomplete Solt-Farber samples, but the former was not despite its well documented high levels in oval cells [8]. Given that several peptides corresponding to CK-19 were more abundant in the preneoplastic foci from both the placebo and rapamycin groups, we cannot attribute the apparent lack of its induction in the incomplete Solt-Farber samples to technical issues. It is more likely a function of dilution due to the presence of AAF-suppressed non-preneoplastic hepatocytes in the regions captured from the incomplete Solt-Farber samples.

To assess the differentiation status of cells in our samples, we examined all of the CYPs that were identified in our analyses. There was an overall pattern of lower peptide abundance in the Solt-Farber placebo samples and the incomplete Solt-Farber samples. The effect was less pronounced in the Solt-Farber rapamycin samples. However, there was a particularly marked reduction in peptides derived from P450 2C11 in all three experimental groups. This highly expressed, constitutive, xenobiotic-inducible cytochrome P450 enzyme is a prototypical “negative acute-phase protein” that is downregulated in response to a number of pro-inflammatory factors in rat hepatocytes [35]. Notable exceptions to CYP downregulation was the higher abundance of P450 2C12 and P450 2C13 in the preneoplastic foci. In a study of the effects of PPARα activators on rats [36], these sex-specific enzymes were regulated in a discordant manner. The increased abundance of these two enzymes in the two Solt-Farber groups cannot be attributed to sex-specific regulation downstream from growth hormone signaling [37, 38]. An examination of our microarray data generated using laser-capture microdissection of the same tissues analyzed in the present study [3], did not show significant induction of either P450 enzyme at the level of mRNA expression. Thus, the overabundance of these enzymes may depend on post-transcriptional regulation associated with hepatic pre-neoplasia.

This observation raises a broader question regarding our proteomic data – to what degree do changes in protein abundance reflect changes in gene expression at the mRNA level? An examination of members of the glutathione S-transferase family is informative. A comparison of the present results with microarray data we generated using LCM of the same rat liver carcinogenesis model [3] shows a high degree of concordance. Three glutathione S-transferases, Gstp1, Gstt3 and Gsta5, were upregulated at the mRNA level. All three showed clearly higher abundance at the protein level. Gsta4, previously identified as induced in the Solt-Farber model [30], showed a small degree of induction in both the microarray and proteomic analyses.

By grouping peptides for individual proteins and creating a heat map, we were able to identify a number of proteins that represent potential, new preneoplastic foci markers and, by extension, potential new markers for HCC. Further studies need to be conducted to characterize the expression and possible functional role of these proteins in the pathogenesis of HCC. An examination of the literature did not disclose prior identification of these proteins in the Solt-Farber model, other relevant HCC model systems or in human HCC. However, a comparison with our published microarray data [3] revealed coordinate upregulation at the mRNA and protein levels for nearly all of the novel markers. Among these were aflatoxin B1, aldehyde reductase member 3 and glucose 6-phosphate 1-dehydrogenase. Only one of the potential protein markers was not detected at or above our threshold in the microarray analysis, aldehyde dehydrogenase (family 1, member L1).

The Solt-Farber model of hepatic carcinogenesis, also referred to as the “resistant hepatocyte” model [39], is a model of progenitor cell marker-positive HCC [8]. A significant proportion of HCC in humans is considered progenitor cell marker-positive [40]. It is presumed that cancer in these individuals arises from a combination of hepatocellular injury leading to expansion of the progenitor cell population and transforming mutations in those progenitor cells, or through dedifferentiation of mature hepatocytes. About 55% of small cell dysplastic foci, the earliest premalignant lesion identified in humans, consist of progenitor cells, suggesting that a subset of HCC may have a progenitor cell origin [41]. Although the Solt-Farber model results in progenitor-marker positive HCC, the earliest preneoplastic foci do not express the ubiquitous progenitor cell marker, CK-19 [27]. This observation was interpreted as suggesting that these lesions may result from a loss of functional hepatocyte differentiation during lesion progression.

As noted above, we showed previously that incorporation of short-term rapamycin administration in the Solt-Farber model has a persistent and dramatic effect on foci size. Our intention was to exploit the proteomic profiling of preneoplastic lesions to assess the degree to which rapamycin modifies the phenotype of foci. Our previous studies using gene expression [3] led us to conclude that foci from rapamycin-treated animals more closely resembled normal liver than did foci from animals implanted with placebo pellets. The present proteomic analyses provide more compelling evidence of an inhibitory effect of rapamycin on the loss of functional differentiation of foci. PCA indicated that foci from the rapamycin group were more similar to normal liver than were foci from the placebo group. Our overall conclusion is that rapamycin, while it has a marked effect on foci growth, has a potent and persistent effect of limiting the loss of mature hepatic function of the foci. We found no evidence in support of the possibility that rapamycin administration selects for a population of less well differentiated, rapamycin-resistant cells, a finding that would mitigate against the use of rapamycin and related agents in HCC chemoprevention.

In summary, we have demonstrated the utility of an approach to achieve proteomic profiling of focal lesions from a model of hepatocellular carcinoma. The coupling of LCM and LC-MS/MS allowed us to selectively quantify proteins within focal lesions. We validated our results by examining a set of known HCC markers. A broader examination of our results disclosed a number of potential markers that have not been identified previously. We were able to further characterize the effect of rapamycin on focal lesion development in the Solt-Farber model, concluding that the drug has the salutary effect of inhibiting both growth and loss of functional differentiation of the foci. Finally, we propose that the combination of LCM and unlabeled, MS-based quantitation of protein abundance may be exploited further by using targeted approaches to assess protein post-translational modifications to study cell signaling dynamics. This approach may ultimately lead to novel information regarding the temporal regulation of signaling pathways and the identification of new pathways involved in the pathogenesis of HCC.

## MATERIALS AND METHODS

### Animals

Male Fischer F344 rats (age 5–6 weeks) were obtained from Charles River Laboratories (Wilmington, MA). All animals were housed under standard conditions with access to food and water ad libitum. They were euthanized by exsanguination under isoflurane anesthesia. Animal studies were performed in accordance within the guidelines of the National Institutes of Health and the Rhode Island Hospital Institutional Animal Care and Use Committee.

Hepatic carcinogenesis was induced using a modification [3] of the protocol originally described by Solt and Farber [39]. Briefly, rats were administered a single intraperitoneal injection of DENA. Two weeks later, they were implanted with a time-release 2-AAF pellet. After one week of 2-AAF administration, a 2/3 partial hepatectomy was performed. Animals were euthanized at 70 days post-DENA injection.

At the time of partial hepatectomy, rats undergoing the protocol described above were randomly assigned to one of two experimental groups. Animals in the rapamycin group were implanted with a time-release pellet that administered the drug at a dose of approximately 2.5 mg/kg/day for 21 days. Control animals received a placebo pellet.

Unmanipulated adult, male rats constituted our control group. For a second control group, animals were given 2-AAF followed by 2/3 partial hepatectomy, which induces oval cell proliferation [42]. Each experimental group contained three animals.

### Tissue processing

At the time animals were euthanized, livers were removed, fixed in 10% neutral buffered formalin and processed for routine hematoxylin and eosin staining as previously described [3]. Sections were also stained for GST-P using a method we have described previously [2].

Tissue areas of interest were dissected from FFPE slides using the Arcturus XT™ LCM System (Life Technologies, Grand Island, NY). Sections (7 μm) were stained using hematoxylin diluted 3-fold in ddH_2_O. Pooled, captured areas of approximately 28 μm^2^ constituted one sample for analysis. We studied three biological samples per group. Four LCM samples were prepared from each biological sample. Slides consecutive to those used for LCM were stained for GST-P (Solt-Farber placebo and rapamycin groups) or hematoxylin and eosin (incomplete Solt-Farber model and normal liver) to validate the choice of areas for analysis.

### Sample preparation for proteomic analysis

Each of the 4 technical replicates per biological sample was processed separately for proteomic analysis using the Liquid Tissue® MS Protein Prep Kit (Expression Pathology, Inc.; Rockville, MD, USA). As per the manufacturer's specifications, LCM samples were transferred to a reaction containing 40 μl of lysis buffer. The mixture was centrifuged at 10,000 × *g* for 1 min, and then incubated at 95°C for 90 min. The clear cell lysate was reduced with 45 mM dithiothreitol for 20 min at 60°C and alkylated with 100 mM iodoacetamide for 15 min at room temperature in the dark. After alkylation and reduction, the samples were centrifuged at 10,000 × *g* for 1 min, after which 2 μl of trypsin solution was added to the lysate. This was followed by overnight incubation at 37°C. The sample was cleared by centrifugation at 10,000 × *g* for 1 min and dried using a centrifugal vacuum concentrator.

### LC-MS/MS analysis

LC-MS/MS was performed on a fully automated proteomic technology platform [43–45] that includes an Agilent 1200 Series Quaternary HPLC system (Agilent Technologies, Santa Clara, CA) connected to a Q Exactive Plus mass spectrometer (Thermo Fisher Scientific, Waltham, MA). The dried tryptic peptides were reconstituted in 20 μl of buffer A (0.1 M acetic acid) and 10 μl was injected for each LC-MS/MS analysis. The LC-MS/MS method was one that has been was described previously (26, 27) to which we made minor modifications. Briefly, the peptides were separated through a linear reversed-phase 90 min gradient from 0% to 40% buffer B (0.1 M acetic acid in acetonitrile) at a flow rate of 250 nl/min through an in-house packed, 15-cm long C18 analytical column The electrospray voltage of 2.0 kV was applied in a split flow configuration, and spectra were collected using a top-9 data-dependent method [46, 47]. Survey full scan MS spectra (*m/z* 400– 1800) were acquired at a resolution of 70,000 with an AGC target value of 3 × 10^6^ ions or a maximum ion injection time of 200 ms. The peptide fragmentation was performed via higher-energy collision dissociation with the energy set at 28 NCE. The MS/MS spectra were acquired at a resolution of 17,500, with a targeted value of 2 × 10^4^ ions or a maximum integration time of 200 ms. The ion selection abundance threshold was set at 8.0 × 10^2^ with charge state exclusion of unassigned and *z* = 1, or 6–8 ions and dynamic exclusion time of 30 seconds.

### Mass spectrometry data analysis and quantitation of relative peptide abundance

MS/MS spectra were searched against the UniProt database (UniProt; downloaded 2/1/2013) using the MASCOT algorithm (Matrix Science, Ltd, London, UK). A concatenated database containing 144,156 “target” and “decoy” sequences was employed to estimate the false discovery rate (FDR). Peak lists were generated using Msconvert (ProteoWizard, v. 3.0.5047), with default parameters and MS2Deisotope filter on. The Mascot database search was performed with the following parameters: trypsin enzyme cleavage specificity, 2 possible missed cleavages, 7 ppm mass tolerance for precursor ions, 20 mmu mass tolerance for fragment ions. Search parameters permitted variable modification of methionine oxidation (+15.9949 Da) and static modification of carbamidomethylation (+57.0215 Da) on cysteine. The resulting peptide spectrum matches (PSMs) were reduced to sets of unique PSMs by eliminating lower scoring duplicates. To provide high confidence, the Mascot results were filtered for Mowse Score (> 20). Peptide assignments from the database search were filtered down to a 1% FDR by a logistic spectral score, as previously described [47, 48]. The mass spectrometry proteomics data sets have been deposited to the ProteomeXchange Consortium (http://proteomecentral.proteomexchange.org) via the PRIDE partner repository with the dataset identifier PXD005845.

Relative quantification of peptide abundance was performed via calculation of selected ion chromatogram (SIC) peak areas. Retention time alignment of individual replicate analyses was performed as previously described [49]. Peak areas were calculated by inspection of SICs using in-house software programmed in R 3.0 based on the Scripps Center for Metabolomics' XCMS package (version 1.40.0). This approach performed multiple passes through XCMS' central wavelet transformation algorithm (implemented in the centWave function) over increasingly narrower ranges of peak widths, and used the following parameters: mass window of 10 ppm, minimum peak widths ranging from 2 to 20 seconds, maximum peak width of 80 seconds, signal to noise threshold of 10 and detection of peak limits via descent on the non-transformed data enabled. When centWave failed to identify an MS peak, the getPeaks function available in XCMS was applied to integrate a pre-defined region surrounding the maximum intensity signal of the SIC. SIC peak areas were determined for every peptide that was identified by MS/MS. In the case of a missing MS/MS spectrum for a particular peptide in a particular replicate, the SIC peak area was calculated according to the peptide's isolated mass, and the retention time calculated based on retention time alignment. A minimum SIC peak area equivalent to the typical spectral noise level of 1000 was required of all data reported for label-free quantitation. Individual SIC peak areas were normalized to the peak area of the exogenously spiked synthetic peptide (DRVpYIHPF) standard added prior to reverse-phase elution into the mass spectrometer.

### Statistical analyses

PCA was performed using the *prcomp* function in R [50] (https://stat.ethz.ch/R-manual/R-devel/library/stats/html/prcomp.html). Venn diagrams were prepared using freeware from the Pacific Northwest National Laboratory (http://omics.pnl.gov/software/venn-diagram-plotter) and VENNY 2.0 (bioinfogp.cnb.csic.es/tools/venny/). To detect peptides that showed significantly different abundance among the experimental groups, technical replicates were averaged for each of the three biological replicates per group. The resulting data were subjected to one-way ANOVA across all samples for each peptide using the *limma* function in R [51]. Volcano plots used adjusted *p*-values derived from pair-wise ANOVA results and fold-change for peptide peak areas. Heat maps were generated using the conditional formatting function in Microsoft Excel. The input for IPA^®^ (Qiagen, Redwood City, CA) was comprised of proteins represented by at least two peptides whose abundance showed a significant difference, based on ANOVA results, between particular experimental groups. IPA categories were considered significant when *p*-values were below the lowest of those obtained for the 5 control datasets [52].

## SUPPLEMENTARY MATERIALS TABLES









## Abbreviations

mTOR: mechanistic target of rapamycin
HCC: hepatocellular carcinoma
LCM: laser capture microdissection
mTORC1: mTOR complex 1
FFPE: formalin-fixed, paraffin-embedded
LC-MS/MS: liquid chromatography-tandem mass spectrometry
DENA: diethylnitrosamine
2-AAF: 2-acetylaminofluorene
COV: coefficient of variation
PCA: principal component analysis
ANOVA: analysis of variance
IPA^®^: Ingenuity Pathway Analysis
CK-19: cytokeratin 19
CYP: cytochrome P450 enzyme
GST-P: glutathione S-transferase placental type
FDR: false discovery rate
PSM: peptide spectrum match
SIC: selected ion chromatogram
