# Supplementary material for: Proteomic analysis of laser capture microdissected focal lesions in a rat model of progenitor marker-positive hepatocellular carcinoma
Source: Oncotarget. 2017 Feb 9;8(16):26041–56. doi: 10.18632/oncotarget.15219 (PMC5432236; doi:10.18632/oncotarget.15219)
Supplement: Supplementary file 1 [file oncotarget-08-26041-s001.pdf]

Proteomic analysis of laser capture microdissected focal lesions in a rat model of progenitor marker-positive hepatocellular carcinom

Supplementary Materials

Supplementary Table 1: All proteomic data. See Supplementary_Table_1

Supplementary Table 2: Significant peptides. See Supplementary_Table_2

Supplementary Table 3: IPA results, including proteins that contributed to the identification of significant canonical pathways. See Supplementary_Table_3
